# Progress in Medicine

**Published:** 1895-11-02

**Authors:** 


### Progress in Medicine.

### GRAVES' DISEASE (*Continued*).

**Acromegaly and Graves' Disease.**—Joffroy[27] refers to a case of acromegaly with slight tachycardia, but without goitre or exorbitis, which was treated by ingestion of thyroid glands of sheep with resulting thyroid intoxication, but no exophthalmos. Murray[28] records three cases of acromegaly. In the first, a male, age 34, there was no symptom of Graves' disease, but after thyroid extract was given for some weeks the languor had diminished and neuralgic pains disappeared. The second was a woman of 34, who also exhibited glycosuria, and a pulse of 100-132. The patient subsequently died, and considerable hypertrophy of the pituitary and thyroid glands and a persistent thymus were found. Murray reminds us that polyuria is common, and diabetes has been twice observed by Marie and once by Strumpfel in acromegaly. Lanceraux[29] relates a case of acromegaly with headache and mental dulness, who also presented all the symptoms of exophthalmic goitre, and also developed hæmorrhoids, polydipsia, polyuria and polyphagia, glycosuria and albuminuria. He considered that the evidence in favour of the group of symptoms being all the effect of some general disturbance was overwhelming. He further remarks that the same symptomatic complex has been met with in other instances, viz., in 1877, by Henrot, in a male aged 36, who had acromegaly, Graves' disease, and diabetes. The heart and thyroid gland were hypertrophied and the pituitary body was replaced by an ovoid tumour the size of a hen's egg. While the nervous system was diseased at various points, three ossiform patches were found in the thickness of the spinal meninges, the pineal gland was twice as large as usual, and the pneumogastric and glossopharyngeal nerves, a portion of those of the brachial plexus, and all the nerves and ganglia of the sympathetic were larger than normal. Another case reported in 1893 by Valat suffered from acromegaly with Graves' disease. Lanceraux remarks that as acromegaly usually precedes hypertrophy of certain glands, viz., the pituitary, thyroid and thymus, and that consequently it is impossible for the affection to be caused by the hypertrophy of these glands. Joffroy's case of acromegaly, aggravated by thyroid gland ingestion, has been alluded to above. Cunningham[30] cites Rogowitsch's experiments, which showed that after the thyroid has been removed in rabbits enlargement of the pituitary body occurs.

**Diabetes and Graves' Disease.**—Glycosuria has been observed in many cases, but it must be remembered that the urine is often not examined for sugar in Graves' disease, or acromegaly. We have already alluded to three cases of acromegaly, Graves' disease, and glycosuria, viz., Murray's, Lanceraux', and Henrot's. Denning[31] records observations on the effect of metabolism of thyroid treatment and mentions glycosuria as not uncommon.

**Myxœdema and Graves' Disease.**—Baldwin[32] records four cases of Graves' diseases which had eventuated in myxœdema. Oppenheimer[33] records cases of sisters, one having Graves' disease and one myxœdema, and refers to Maude's[34] cases as the only other similar instance he can find recorded. Babinski[34] has on two occasions observed the coincidence of Graves' disease and symptoms of myxœdema in the lower limbs, and Hektoen[35] has seen the same group of symptoms in one case.

**Lesions of the Nervous System and Graves' Disease.**—Brissaud[36] cites as among the distinctly nervous diseases which may be primarily or secondarily associated with exophthalmic goitre, are epilepsy, hysteria, chlorosis, tabes dorsalis, syringomyelia, scleroderma, chorea, and insanity, and refers to the experiments of Filehne and Durdufi, who, having resected the restiform bodies in young rabbits, found the operation to be followed immediately by exophthalmia, swelling of the thyroid gland, and tachycardia. Oppenheim[37] also cites the fact that Filehne obtained goitre at times on electrical stimulation, and that Durdufi, having incised the tub. acustica, got Stellwag's sign; while Bienfalt practically confirmed Filehne's experiments. Further, several observers have found more or less hæmorrhage in the floor of the fourth ventricle. Hale White noted an old superficial hæmorrhage, extending from the middle line to the corpus restiforme, and involving the sixth nucleus on one side. Mendel found one restiform body smaller than the other, and the right solitary fasciculus atrophied. Oppenheimer goes on to say that at the time Mendel's paper was presented, Oppenheim remarked that the corpora restiforma are normally so unlike that a slight difference in the size would have but little weight. In most autopsies, however, gross lesions of the central nervous system have not been observed.

A case of Graves' disease, with monocular symptoms and unilateral thyroid hypertrophy, is recorded by Fridenberg.[38] A married lady, age 24, who had a great deal of worry, came with distinct exophthalmos and Graves' symptom in the left eye, insufficiency of the internal recti, venous pulsation on left disc, the right eye was normal. There was some flushing of the face, most marked on the left side, and greatly increased by exertion or mental excitement. The thyroid was not noticeably enlarged, but on palpation increase of right lobe and isthums was made out. Fine tremor of tongue and hands, five to six to the second. Heart was enlarged and tumultuous. Excluding doubtful cases, the author found that of thirteen cases the right eye was alone affected in ten. Of the three cases involving the left eye, but one, that of Burney Yeo was associated with hypertrophy of the opposite lateral lobe of the thoroid, and in this case both lobes eventually became much enlarged. Ocular and thyroid symptoms were limited to the right side in Chvostek's case only. In eight the unilateral exophthalmos was associated with general enlargement of the thyroid.

Finally, reference must be made to recent reports of reflex cases. Scanes Spicer[39] refers to the case of a neurotic young woman who came to him for the removal of nasal polypi. The thyroid gland presented a thrill and pulsation, there was tachycardia and fine tremor of arms. These symptoms began simultaneously with the symptoms of nasal polypi three years previously. After removal of the polypi the other symptoms improved. Stoker[40] referred to the case of a man who had nasal polypi and a soft goitre, which latter had resisted all treatment until the galvano-cautery was applied to the polypi. The goitre lessened, and with the continuance of the treatment the goitre disappeared in two months. Cases[41] of cure of Graves' disease by operation on the nose are reported by Hofmann, Hack, and Fränkel. On the other hand that many cases have had their nasal passages treated without any result whatsoever on the disease.

Hürthle, as the result of a series of experiments on dogs, states that the thyroid gland cannot be stimulated to secretion by stimulation of its nerves, and that the statesof the blood must supply the necessary stimulus. Eulenberg[42] states that ligature of the bile ducts has been shown to lead to an increase in the thyroid secretion.

**Relation of Graves' Disease to Rheumatism.**—H. Mackenzie[43] observes that quinsy and rheumatism are antecedent or coincident in a significant number of cases. Out of some forty cases he has noted quinsy in nine and acute rheumatism in five; this agreeing with the evidence of others. S. West records two cases, and remarks that they present two features of interest, viz., that they occurred in sisters and that in each there was a history of rheumatic fever and heart lesion. In a previous report of fifty-six cases, rheumatic fever had occurred in 11 per cent.

Heredity seems in many cases to have an undoubted influence on the occurrence of Graves' disease. Thus we have already alluded to two sisters, one with Graves' disease, the other with myxœdema, reported by Oppenheimer. In H. Mackenzie's forty cases five had a family history with exophthalmic goitre. West's two cases were sisters. Ball, cited by Oppenheimer, reports a case of a mother and two sisters, and Œsterreicher, also cited by the same writer, has recorded a remarkable instance[44] of a hysterical woman who had ten children, eight of whom had exophthalmic goitre. Maude[45] reports a woman with myxœdema, whose daughter had exophthalmic goitre. Progressive muscular atrophy, in association with Graves' disease, is reported by Bathurst,[46] though Beevor thought it might possibly be a case of pseudo-hypertrophic paralysis.

Bryson's symptom in Graves' disease has been investigated by Patrick,[47] who found the average chest expansion in forty cases to be 4·3 centimetres (hand grasp 43·75 kilog.), in twenty-eight other chronic diseases 4·8 per cent. (grasp 56·36 kilog.)—that is, the expansion in Graves' disease is diminished half a centimetre (the grasp 12·61 kilog.). Thus the chest expansion is diminished $10\frac{1}{2}$ per cent., and the hand grasp $22\frac{2}{5}$ per cent., the grasp being diminished more than twice as much as the expansion, and thus the idea that the diminished chest expansion is simply part of a diminished vitality or energy is borne out both by these figures as well as by the history of the individual cases. He believes that the diminished power of conveyance often observed, and the occasional affection of the laryngeal muscles, is quite analogous to these other findings, and is simply a part of a general myasthenia. The value of the Bryson sign thus loses its importance as a diagnostic sign of Graves' disease.

**Operative Treatment.**—The recent contributions to the treatment of Graves' disease are chiefly in connection with the question of surgical operations on the thyroid gland. We find reports of the following cases amongst recent literature :—

| No. of Case. | Reporter. | Sex. Age. | Duration of Disease. | Main Symptoms. | Operation. | Result. |
|---|---|---|---|---|---|---|
| 1 | McCosh[48].. | F. 20 | 3 years | Exophthalmos marked, thyroid large, especially right lobe, palpitation, pulse 150, very nervous and anæmic | Thyroidect. of R. lobe 3 months previously | For 2 weeks pulse rapid, sometimes 200. The protrusion of eyes diminished notably in 12 hours; finally disappeared; left half decreased to half its former size. Pulse averaged 115. (Note.—This case had been treated for 2 months without effect by galvanism, arsenic, and thyroid extract.) |
| 2 | Curtis[49] ... | 1st case | — | Graves' disease. Pulse 120 | Thyroidect. R. lobe | Improvement. Seen a year later, improvement had remained, though there was still some exophthalmos. Pulse then 80 to 100. Hysterical and nervous symptoms had disappeared, also insomnia |
| 3 | ,, ... | 2nd case | — | Graves' disease | Thyroidect. R. lobe, October, 1894 | Great improvement |
| 4 | ,, ... | 3rd case | — | Goitre, rapid pulse, nervousness, no albumen in urine | Thyroidect. R. lobe, October, 1894 | Died. Apparently with symptoms of hyperthyroidation |
| 5 | Gerster[50]... | F. 24 | 1½ years | Typical Graves' symptoms, rapid pulse, 122-146, thyroid much enlarged, especially right lobe, proptosis especially on right side. Dyspnœa on exertion from compression | Thyroidect. R. lobe, December 3, 1890 | Dec. 5, pulse 106-136; Dec. 6, pulse 102-116; Dec. 8, pulse 84-96; Dec. 9, pulse 78-92. From Dec. 9 the exophthalmos was notably diminished. Improvement gratifying and prompt. |
| 6 | Tuffier[51] ... | F. 27 | 7 years | Cystic enlargement of right lobe, with exophthalmos and other usual symptoms | Partial thyroidect. | All Graves' symptoms and exophthalmos entirely disappeared. Cure. Iodine injection, tapping, and electrical treatment tried without benefit |
| 7 | Edmunds[52] | 1st case | — | Graves' disease. No details | Partial thyroidect. | Improved. No details |
| 8 to 19 | Mikulicz*[53] | 11 cases | — | All presented marked tachycardia, and 5 serious dyspnœa | In 2 cases ligation of arteries, in 9 extirpation | 6 completely cured, 4 considerably improved, 1 slightly improved (unilat. ligation). In one case after unilateral resection, temporary improvement, with subsequent hypertrophy of the opposite lobe and recurrence of symptoms. After this lobe was removed result was satisfactory |
| 20 to 27 | Krönlein[54]. | — | — | Graves' disease | Resection | All much improved. Exophthalmos remained in one case |
| 28 to 35 | Lemke[55] ... | — | — | Well-marked Graves' disease | Partial resection | All survived, and in all the more important symptoms were relieved, viz., exophthalmos, goitre, nervous agitation, and tremor |
| 36 | Petersen[56]. | F. 34 | — | Symptoms 7 years. For some time before operation pulse 120, proptosis. No V. Græfe symptom. Tremor, flushing, hyperidrosis, and goitre present | Resection left lobe, 1889 | All the symptoms greatly improved, and 5 years after operation she had no proptosis, no tremor, no tachycardia, no goitre, and she was quite well, except that the pulse remained at 90-100. Digitalis and galvanism had been tried without effect |
| 37 to 75 | Kocher[57]... | — | — | Vascular goitre with symptoms of Graves' disease | In 34 either ligation or partial extirpation. He prefers the former unless extreme dyspnœa exists. He usually ligatures 3 arteries | 3 have since died; all the rest either cured or greatly improved |

* Mikulicz remarks that the effects seem to be the same whether ligature, enucleation, or resection is performed, and that in some cases Graves' disease has disappeared after simple puncture or incision of a simple goitre.

[28] Brid. Med. Journ., Feb. 9, 1895. [29] Med. Week, 1895, p. 109. [30] Coc. cit. [31] Munch. Med. Woch., April 23, 1895, Epit. B. M. 7, May 25. [32] Lancet, Jan. 19, 1895. [33] Journ. of Nerv. and Ment. Dis., April 1895. [34] Barth. Hosp., Sep. 1893. [34] Med. Week, 1895, p. 394. [35] Loc. cit. [36] Loc. cit. [37] Loc. cit. [38] Med. Rec., July 13, 1895. [39] B. M. J., Nov. 19, 1894; Journ. of Laryng., Feb., 1895. [40] Ibid. [41] Cited by Oppenheimer, Journ. of Nerv. and Ment. Dis, April, 1895, p. 220. [42] Arch. f.d.ges. Phys. Bd. 56. [43] Lancet, Sept. 30, 1890. [44] Lancet, May 18, 1895. [45] St. Barth. Hosp. Rep., 1893. [46] Lancet, Aug. 31, 1895. [47] N. Yk. Med. Journ., Feb. 9, 1895. [48] Med. Rec., Aug. 24, 1895. [49] Ibid. [50] Annals of Surgery, Mar., 1895. [51] Lancet, June 1, 1891. [52] Brit. Med. Journ., May 25, 1895. [53] Med. Week, Ap. 26, 1895. [54] Ibid. [55] Med. Press and Circ., Jan. 30, 1895. [56] N. Y. Med. Journ. [57] Med. Week, Mar. 1, 1895.

